# A Reproducible Digital Workflow for Patient-Specific 3D-Printed Teeth with Anatomically Stratified Enamel and Multi-Material Caries for Preclinical Operative Dentistry

**DOI:** 10.3390/jfb17080355

**Published:** 2026-07-24

**Authors:** Alexandru Mihai Micu, Robert Mihai Bacalu, Teodor Raul Constantin, Alexia-Ecaterina Cârstea, Lucian Toma Ciocan, Vlad-Gabriel Vasilescu, Bogdan Dimitriu, Mihaela Pantea, Silviu-Mirel Pițuru, Marina Imre

**Affiliations:** 1 Faculty of Dentistry, “Carol Davila” University of Medicine and Pharmacy, Dionisie Lupu Street, No. 37, District 2, 020021 Bucharest, Romania; alexandru-mihai.micu0720@stud.umfcd.ro (A.M.M.); robert-mihai.bacalu0720@stud.umfcd.ro (R.M.B.); teodor-raul.constantin2022@stud.umfcd.ro (T.R.C.); 2 Discipline of Dental Prosthesis Technology, Faculty of Dentistry, “Carol Davila” University of Medicine and Pharmacy, Dionisie Lupu Street, No. 37, District 2, 020021 Bucharest, Romania; vlad.vasilescu@umfcd.ro; 3 Department of Endodontics, Faculty of Dentistry, “Carol Davila” University of Medicine and Pharmacy, 8 Eroilor Sanitari Blvd, 050474 Bucharest, Romania; bogdan.dimitriu@umfcd.ro; 4 Department of Prosthodontics, Faculty of Dentistry, “Carol Davila” University of Medicine and Pharmacy, Dionisie Lupu Street, No. 37, District 2, 020021 Bucharest, Romania; mihaela.pantea@umfcd.ro (M.P.); marina.imre@umfcd.ro (M.I.); 5 Department of Professional Organization and Medical Legislation-Malpractice, “Carol Davila” University of Medicine and Pharmacy, Dionisie Lupu Street, No. 37, District 2, 020021 Bucharest, Romania; silviu.pituru@umfcd.ro

**Keywords:** dental education, additive manufacturing, 3D printing, MSLA, PolyJet, phantom head, operative dentistry, simulation, digital workflow, multi-material printing

## Abstract

Conventional typodonts for preclinical operative dentistry are idealised and lack the variability and tactile feedback of natural teeth; virtual-reality and haptic simulators remain costly for large cohorts. This study developed a reproducible, low-cost digital workflow for patient-specific, multi-material 3D-printed dental simulators and evaluated the models from a learner’s perspective. Intraoral and CBCT data from a single de-identified case were co-registered to a Virtual Patient. A modular tooth-socket architecture with trans-apical screw retention allowed repeatable individual tooth replacement; each tooth combined a variable-thickness enamel shell over a dentin core with multi-material carious lesions. Two fabrication routes (MSLA and PolyJet) and an adopted hybrid route (MSLA base, PolyJet teeth) were compared for cost, time and fidelity. The hybrid produced complete bimaxillary models at low consumable cost while preserving the source occlusion and contacts. Fifty dental students evaluated a PolyJet specimen hands-on: didactic utility scored highest (4.32 ± 0.65 on 1–5) and visual realism exceeded tactile realism (*p* < 0.001); 94% rated it at least comparable to a natural extracted tooth and 62% preferred it, while perceived hardness remained the main limitation. The workflow enables scalable in-house production of patient-specific simulators, with tactile realism the priority for material development.

## 1. Introduction

Dental education requires the acquisition of fine psychomotor skills under conditions in which patient exposure to learner error is ethically unacceptable [1]. Simulation-based preclinical training is therefore the central pillar of the operative dentistry curriculum, corresponding to Miller’s “Shows How” tier of clinical competence [2,3]. The didactic substrate on which students rehearse restorative procedures has evolved from extracted natural teeth (historically the gold standard for tactile realism, but increasingly constrained by limited and unpredictable availability, by cross-infection risk and the biosafety burden of handling and sterilising human tissue, and by the impossibility of standardisation inherent in biological variability [4,5,6,7]) to industrially produced typodonts (Frasaco, Nissin, Columbia) and, more recently, to virtual reality, haptic and augmented-reality simulators [8,9,10].

Each of these alternatives presents trade-offs. Conventional typodonts offer reproducibility and standardised assessment, but the anatomy is excessively idealised: arch forms are symmetric, contact points perfect, and pathology either absent or represented as flat colour marks on a homogeneous monomaterial substrate. Virtual reality and haptic systems eliminate physical consumables but remain tactilely inferior to physical substrates and prohibitively expensive for large undergraduate cohorts [8]. Additive manufacturing occupies an intermediate position: it can deliver both the anatomical realism of natural teeth [11] (because the geometry is derived directly from real patient scans) and the standardisation and replicability of industrial models, while maintaining a per-unit cost compatible with consumable status [12].

Several groups have reported 3D-printed educational models for restorative training [13,14,15,16,17], and a recent narrative review documents their expanding role across dental specialities [18]. These designs span anatomically realistic teeth reconstructed from individual patient datasets [19,20], teeth carrying simulated carious lesions for cavity-preparation and selective-caries-removal exercises [21,22,23], and printed teeth with separate enamel and dentin layers for crown-preparation training [24]. Most recently, multi-coloured 3D-printed teeth produced via PolyJet technology have been explored for dental education: Lugassy et al. [16] demonstrated augmented visual feedback through coloured layer systems for cavity preparation training, while Dosch et al. extended the multi-material principle to integrated enamel, dentin, pulp, carious dentin, and restorative materials within a single printed tooth, with favourable pilot evaluation among clinical students [25]. These contributions have collectively validated the feasibility of digital-to-physical pipelines for dental education and established multi-material stratification as a viable approach to improving tactile and chromatic fidelity. However, published workflows typically share one or more of the following limitations: (i) reliance on monolithic, single-material prints that preclude individual tooth replacement when damaged; (ii) anatomically simplified roots and arbitrary retention geometries that do not reproduce realistic intra-alveolar stability; and (iii) caries represented through single demonstration specimens rather than through a systematic curricular library spanning the full range of Black classes, restorations with secondary pathology, and non-carious substance losses.

The aim of this study was to develop a standardised workflow for patient-specific, multi-material 3D-printed dental simulators and fabricate complete bimaxillary models. The objectives were to develop a modular replaceable tooth system, create anatomically stratified teeth with representative dental defects, compare MSLA, PolyJet, and hybrid manufacturing workflows, and evaluate the educational realism and value of the PolyJet-printed models.

## 2. Materials and Methods

### 2.1. Study Design

**Figure 1:**
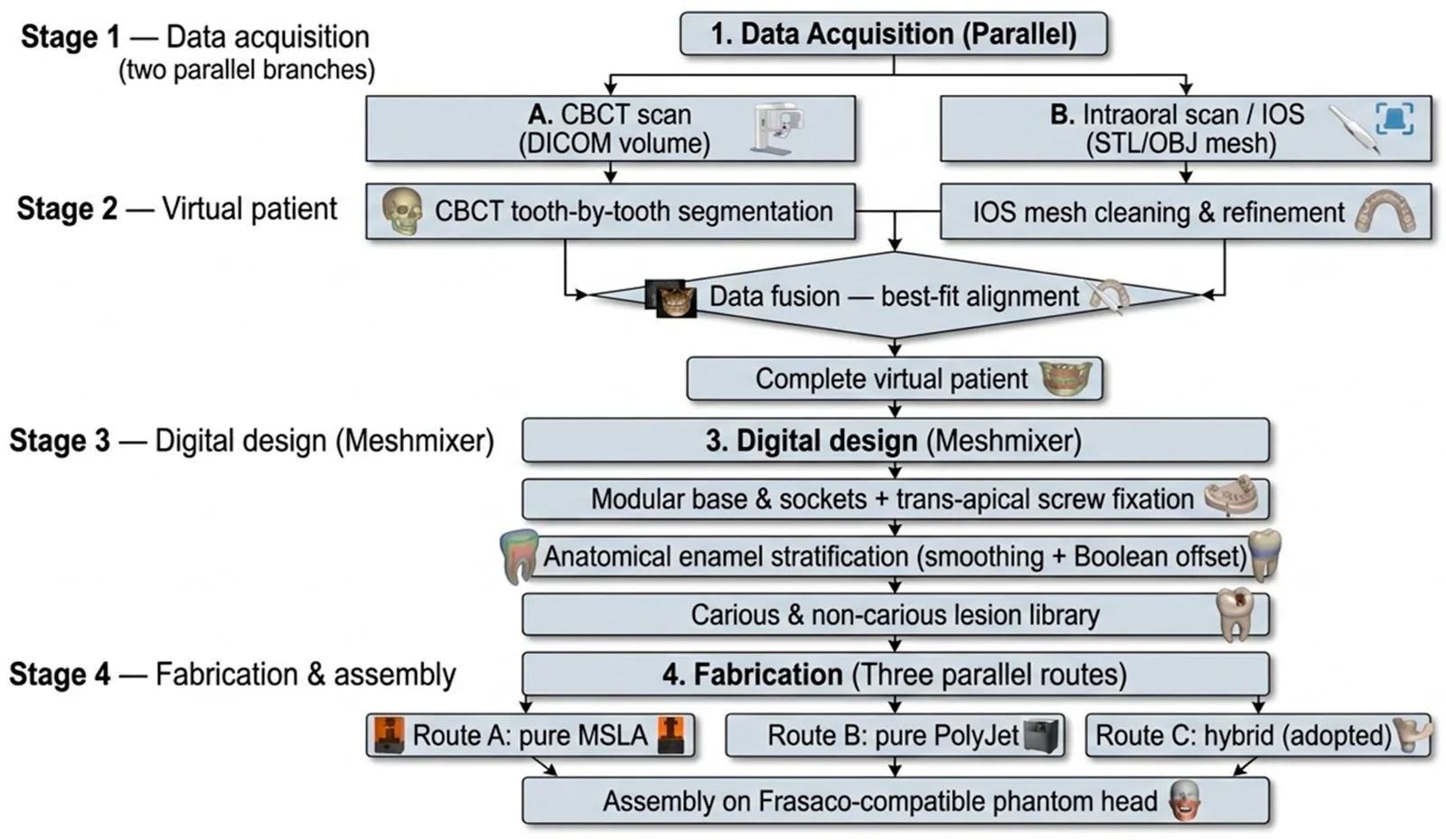
Overview of the digital workflow.

This study comprised two sequential components. In the first, a standardised digital workflow was developed for the design and fabrication of patient-specific, multi-material 3D-printed dental simulators, and three complete prototypes were produced via two parallel fabrication routes and an adopted hybrid route (Section 2.2, Section 2.3, Section 2.4, Section 2.5, Section 2.6, Section 2.7, Section 2.8, Section 2.9 and Section 2.10). In the second, the perceived fidelity and didactic value of the resulting PolyJet-printed stratified tooth were evaluated through a cross-sectional questionnaire study among dental students (Section 2.11). The development workflow and the evaluation methodology are reported separately below (Figure 1).

### 2.2. Source Data and Ethical Considerations

The imaging data used as the anatomical substrate for the MK1 prototype. Both the intraoral scan and the CBCT examination had been acquired previously for routine diagnostic and treatment-planning purposes unrelated to the present study; consequently, no additional imaging and no additional ionising radiation exposure was incurred for the purpose of this work. The data were fully anonymised at source and were handled in compliance with the General Data Protection Regulation (GDPR) [26]. The patient provided written informed consent for the secondary use of their de-identified imaging data for research and educational purposes. The study was reviewed and approved by the Ethics Committee of “Carol Davila” University of Medicine and Pharmacy, Bucharest, Romania (approval NO. 11717, 8 May 2026). [27].

The case was selected because it met the criteria of a versatile didactic substrate: (i) complete dental arches with stable occlusion and physiological contact points; (ii) high-quality, artefact-free CBCT and full-arch colour IOS; and (iii) representative coronal morphology suitable for foundational undergraduate training [12].

### 2.3. Acquisition and Integration of the Patient Data

The intraoral scan was acquired with a Medit i700 scanner (Medit, Seoul, Republic of Korea) and processed in Medit Link/Medit Model Builder/Medit Design (Medit, Seoul, Republic of Korea) [28,29,30,31]. The in vitro accuracy of the Medit i700, assessed under standardised ISO 20896-1 [31] conditions, has been characterised previously, with consistent trueness and precision reported for this scanner [32], supporting its use as the source-data acquisition device for the present workflow. The CBCT examination had been acquired with a MyRay Hyperion X9 pro hybrid PAN/CEPH/CBCT unit (Cefla S.C., Imola, Italy) operating at 90 kVp and 5 mA, with a 7.2 s exposure (CTDIvol 2.4 mGy, dose-length product 43.4 mGy·cm); the volume was reconstructed at a 0.3 mm isotropic voxel size over a 16 × 18 cm field of view. After verification of image quality and absence of artefacts in 3D Slicer (version 5.4.0, open-source software) [33], the CBCT data were exported as DICOM and segmented in Blue Sky Plan (Blue Sky Bio, Libertyville, IL, USA) [34,35,36,37]: density thresholding was used to isolate hard dental tissues from alveolar bone, and a semi-automatic tooth-by-tooth segmentation produced an STL conglomerate preserving original spatial relationships.

**Figure 2:**
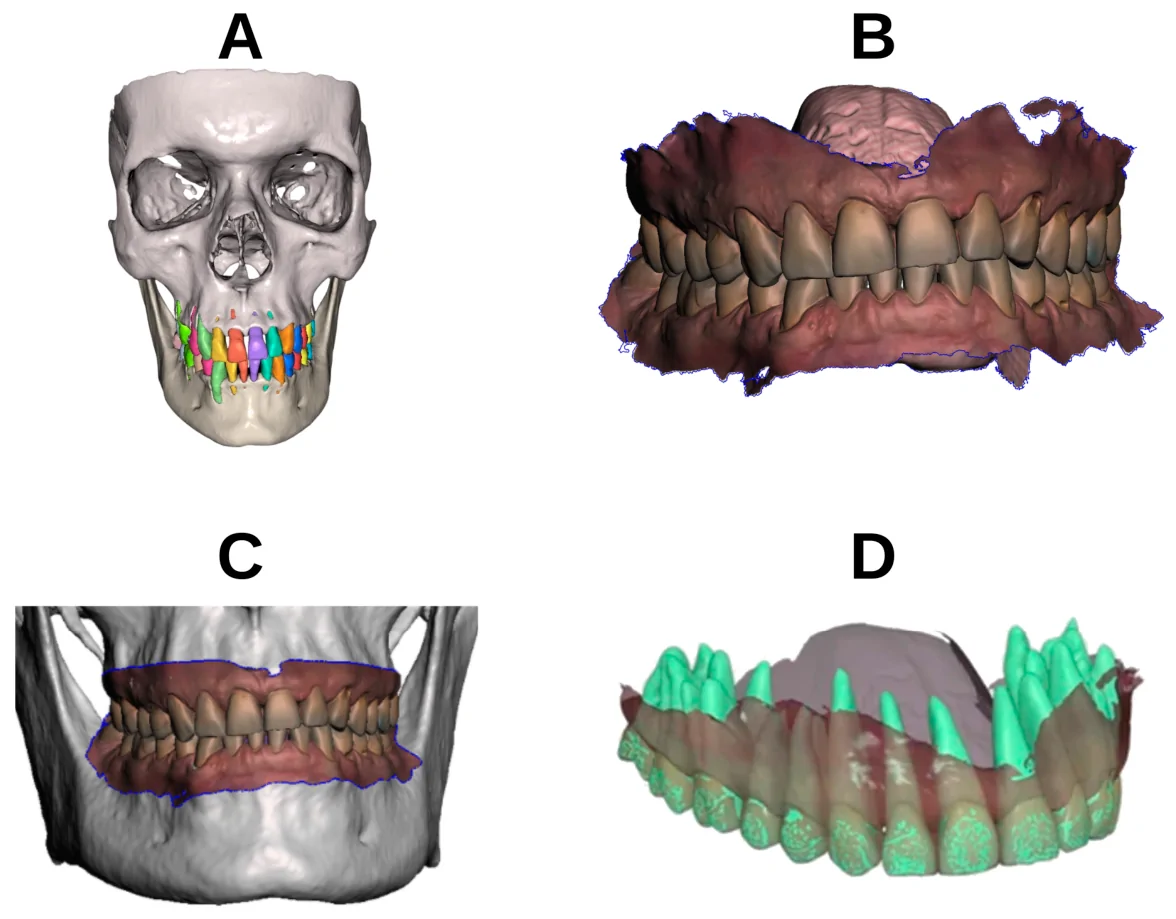
Patient-specific data acquisition and virtual patient generation. (**A**) CBCT volume with semi-automatic tooth-by-tooth segmentation, colour-coded per tooth. (**B**) Full-arch intraoral scan (IOS) in occlusion. (**C**) Co-registration of the CBCT and IOS datasets. (**D**) Registration accuracy: the segmented CBCT dentition (green) coincides with the optically scanned crown morphology.

The two datasets were co-registered in Medit Design using a manual three-point alignment refined by a Best-Fit algorithm with the IOS as reference [38,39]. Registration accuracy was verified through cross-sectional inspection, ensuring that the long axes of the CBCT-segmented teeth coincided with the optically scanned crown morphology and that the original occlusion was preserved (Figure 2) [40,41].

### 2.4. Geometric Processing in Meshmixer

**Figure 3:**
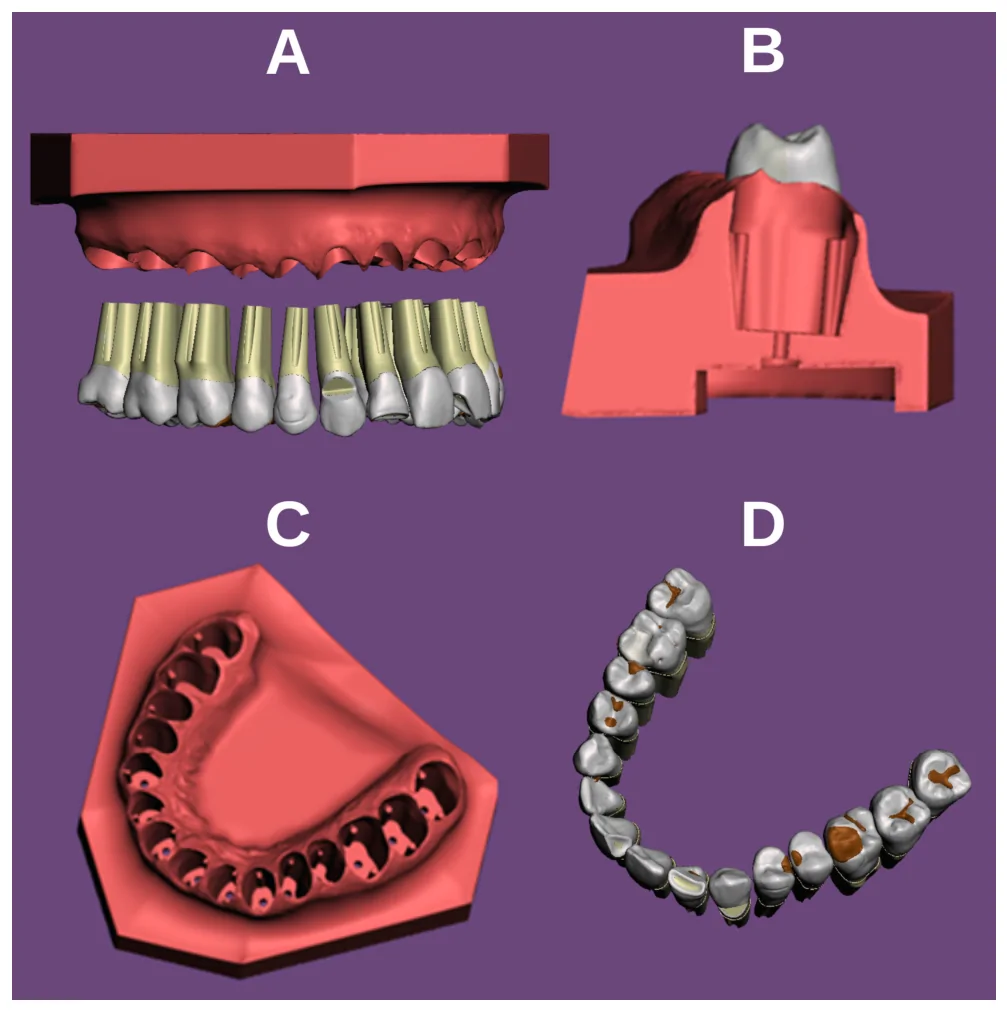
Design of the modular MK1 architecture. (**A**) Exploded view showing tooth-socket correspondence. (**B**) Cross-section through a single socket. (**C**) Base with individual alveolar sockets. (**D**) Complete tooth garniture as independent components.

All subsequent geometric work was performed in Autodesk Meshmixer (Figure 3). (A) The crowns were manually removed from the IOS mesh, the resulting holes were filled, and the gingival surface was regularised by smoothing. An internal recess was added to the underside of the base to accommodate the heads of the trans-apical fixation screws. (B) CBCT-segmented teeth were grouped into frontal, left lateral, and right lateral units to define a common path of insertion. Each group was sectioned a few millimetres apical to the cemento-enamel junction, preserving the cervical emergence profile while eliminating undercuts. The roots were then extruded apically and tapered into a non-retentive truncated-conical geometry ending in a flat apical butt joint. (C) Two parallel cylindrical guides were added to the mesial and distal surfaces of each root, embedded to half their diameter and aligned with the insertion path, preventing rotation while maintaining a single seating trajectory. (D) Sockets were created by Boolean Difference using the teeth as cutting tools. A 0.2 mm radial offset was applied only to the lateral root surfaces, providing passive insertion, compensation for polymerisation shrinkage, and simulation of the gingival sulcus. The flat apical butt-joint surface remained unoffset, creating a rigid zero-tolerance vertical stop that preserves the original occlusal and interproximal relationships by preventing vertical displacement of the tooth. (E) A trans-apical screw-retention system was generated using Boolean operations. Pilot channels (Ø 1.55 mm or 1.3 mm) were drilled through each root, and counterbored access holes with a Ø 5 mm head recess were created in the model base. M1.7 × 8 mm screws were used in most positions, while M1.4 × 6 mm screws were used for mandibular incisors. Socket floors were standardised to 3 mm thickness to ensure adequate screw anchorage. (F) A standardised pentagonal adapter plate with a central M7-threaded cavity was added to the underside of the base for direct compatibility with Frasaco-type phantom heads.

### 2.5. Anatomical Enamel Stratification (Multi-Shell Tooth Architecture)

A defining feature of the present iteration of the model is the anatomically variable enamel shell, generated through a dedicated Meshmixer protocol applied identically to every tooth in the garniture.

Each tooth was processed individually. The surface corresponding to the anatomical crown (coronal to the cemento-enamel junction) was manually selected, and the Meshmixer Smooth function was applied to the selection with a fixed parameter set used identically for all teeth. By construction, the Smooth function displaces each vertex by an amount that increases with its distance from the selection boundary: surface points close to the cervical margin (the selection edge) are displaced minimally, whereas points farther from the margin, on the cuspal tips, incisal edges and central occlusal fossae, undergo progressively larger inward displacement. The result is a volumetrically reduced copy of the tooth in which the crown surface has receded anatomically, with greater recession on the cusps and incisal edges and minimal recession at the cervix.

**Figure 4:**
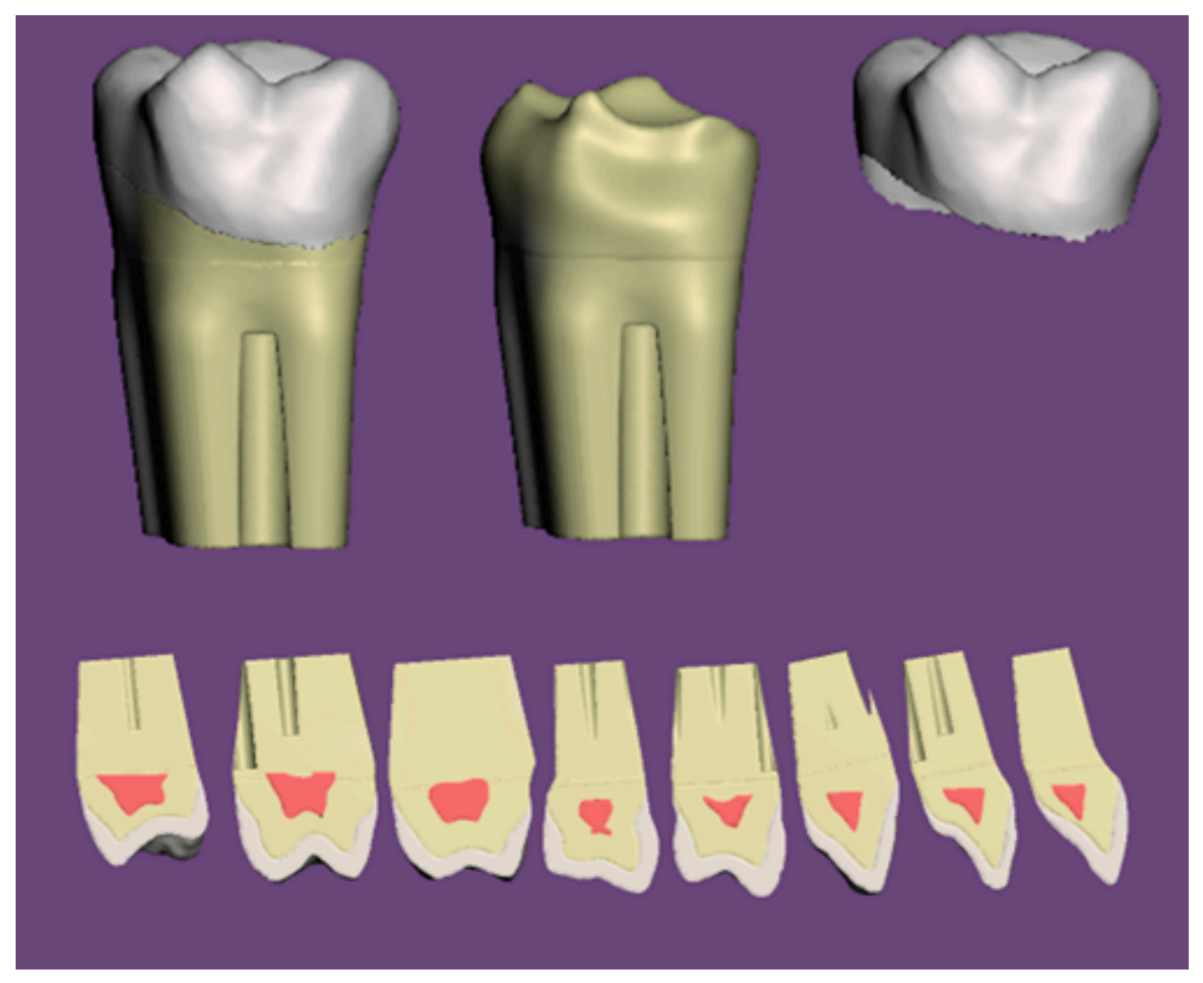
Anatomically variable enamel stratification.

This smoothed volume was treated as the dentin core and was subtracted from the original, undeformed tooth by Boolean Difference, yielding the enamel shell as a separate closed volume. Because the Smooth recession is spatially non-uniform in the way described above, the resulting shell follows an anatomically oriented thickness gradient, qualitatively consistent with natural enamel: thicker on cuspal tips and incisal edges, progressively thinner toward the cervical margin, terminating at the CEJ [42,43]. The same parameter set was used on all teeth, yielding a reproducible protocol that does not require per-tooth manual adjustment (Figure 4). The anatomical orientation of the resulting gradient is therefore an emergent geometric consequence of the smoothing operation rather than a target enforced against the source anatomy; its qualitative correspondence to natural enamel was confirmed by cross-sectional inspection but was not quantified against the CBCT dataset.

Root canal systems were deliberately omitted from the tooth design, because the trans-apical screw occupies the apical third of the root and is mechanically incompatible with a patent canal lumen. Pulp chambers, on the other hand, were modelled, so that students reaching the chamber during excavation receive an explicit visual signal of pulpal involvement.

### 2.6. Design of Carious and Non-Carious Lesions

To ensure complete curricular coverage, the lesion distribution across the two arches included:

- Carious lesions: occlusal, interproximal and cervical caries of varying depth, covering all Black cavity classes;
- Restorations with secondary pathology: pre-existing composite and amalgam restorations, each surrounded by a coloured halo representing marginal infiltration and recurrent caries beneath the restoration margin;
- Non-carious tooth substance loss: advanced attrition and abrasion facets, cervical wedge-shaped (abfraction-type) lesions, and a non-penetrating mesial angle fracture of a maxillary incisor, involving both enamel and dentin but sparing the pulp.

**Figure 5:**
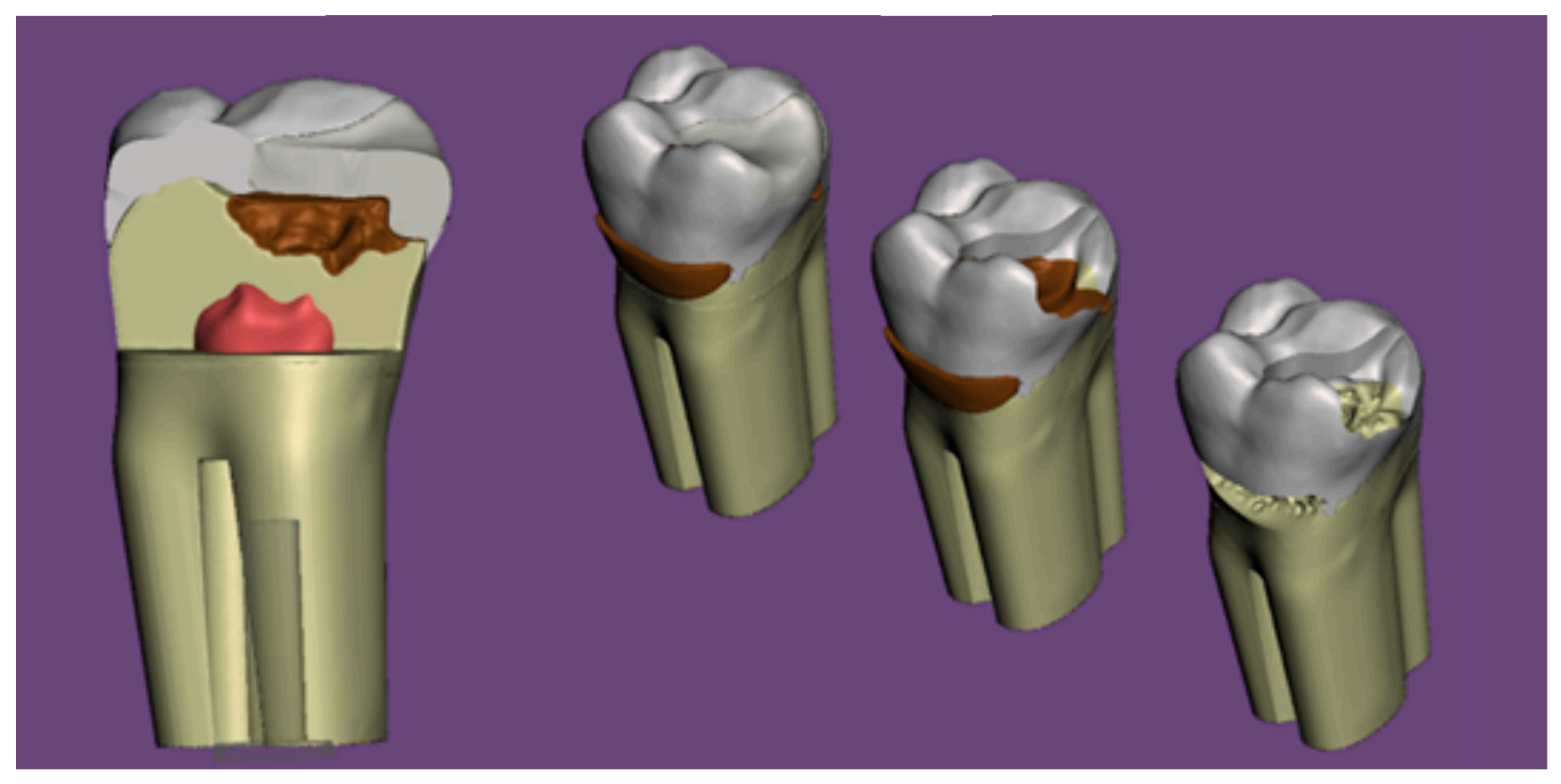
Indirect modelling of lesions and restorations as independent volumes.

Carious lesions were modelled using an indirect method, in which each lesion is built as an independent positive volume (Figure 5). Direct sculpting is destructive, hard to revert, prone to irregular margins, and incompatible with the PolyJet workflow, which requires the external tooth shell to remain topologically intact for multi-material assignment. In the indirect workflow, the lesion body was seeded either from geometric primitives (spheres, cubes) or from duplicated and extruded patches of the tooth surface itself and then manually refined in Meshmixer using sculpting and transformation tools to match the intended clinical shape, extension and margin configuration.

#### Curricular Coverage and Evaluation Rubric

**Figure 6:**
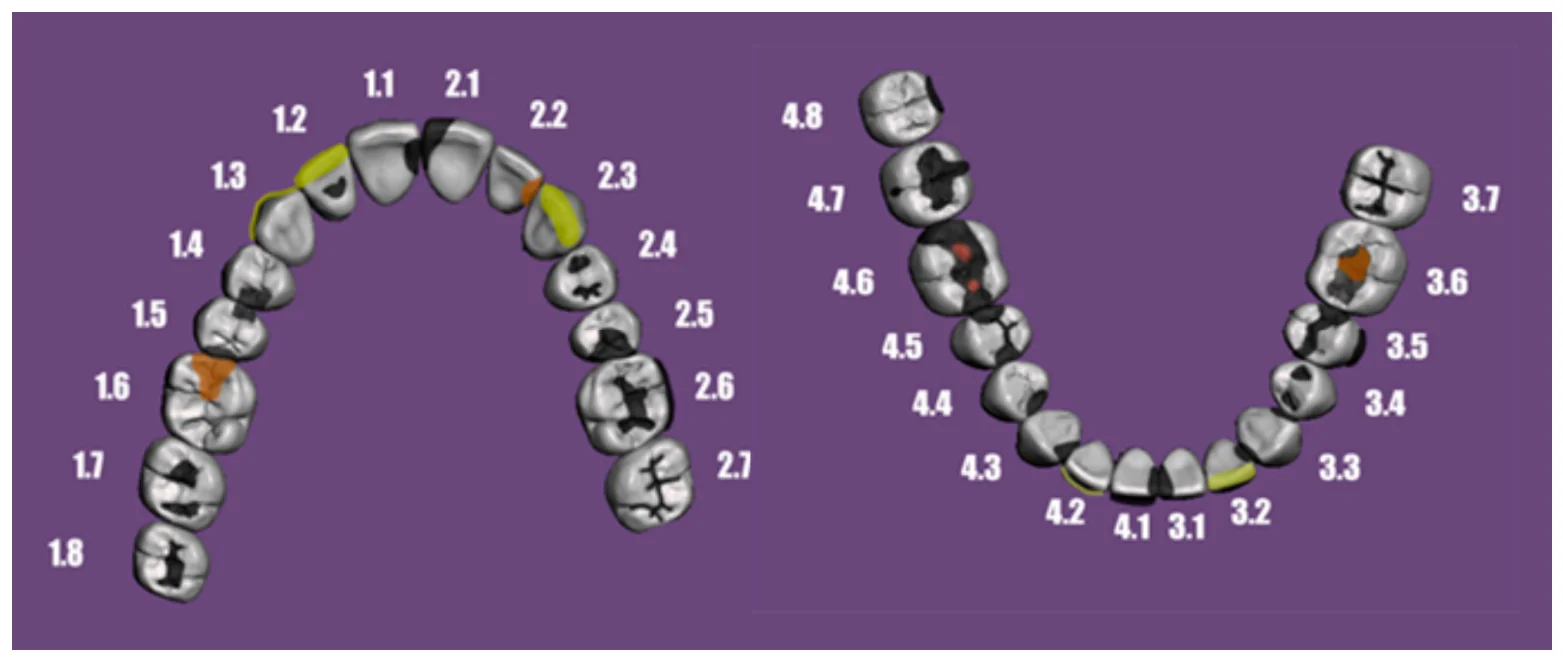
Visual map of the MK1 lesion rubric: maxillary (**left**) and mandibular (**right**) arches in FDI notation. Colour code: black = primary carious lesion; yellow = non-carious lesion (abfraction, abrasion, attrition, trauma); orange = restoration with secondary caries; red = pulpal involvement. Full lesion inventory in Table S1.

The lesion distribution was designed against an explicit curricular target: the full set of procedures typically covered in a single semester of undergraduate Operative Dentistry teaching at our faculty. A written and visual grading rubric was produced in parallel with the digital design, in which every tooth of the garniture is individually documented together with the lesion(s) it carries, the Black class or non-carious category of each lesion, the expected preparation, and the didactic objective associated with that tooth. Because every student in a cohort receives the same lesions on the same anatomy, the rubric also serves as a standardised grading instrument. In the MK1 prototype, this rubric distributes 34 documented lesions across the 30 teeth of the garniture, four teeth carrying two lesions each. They comprise 24 primary carious lesions across all five Black classes, 3 restorations with recurrent caries, 1 lesion with pulpal exposure, 1 mesial-angle incisor fracture, and 5 non-carious substance-loss lesions. The full inventory by clinical category and FDI position is given in Table S1, and mapped by arch in Figure 6.

### 2.7. Fabrication Route A: MSLA Workflow

In the pure MSLA route, bases were printed on a Phrozen Sonic Mini 8K S (Phrozen Technology, Hsinchu, Taiwan) MSLA system [44] using a standard ABS-like resin (Anycubic, Shenzhen, China) to minimise cost, at a print orientation of 30–45° on medium-thickness supports with spherical contact points.

Tooth garnitures were printed using NextDent C&B MFH (NextDent, Soesterberg, The Netherlands), a Class IIa biocompatible crown-and-bridge resin [45]. Teeth were oriented with the flat apical butt-joint surface parallel to the build plate, so that the support structures attached exclusively to the apical surface and the occlusal relief remained untouched by support contact points. This orientation is critical to preserve the morphological fidelity of the cuspal tips and fissure pattern, which are the regions the student will subsequently manipulate.

Post-processing followed solvent cleaning protocols specific to each material: tooth components were rinsed in two successive baths of 99% IPA, with immersion limited to 3 min for the NextDent material to prevent surface degradation, followed by support removal and final UV/thermal post-curing (30 min for NextDent teeth, 7 min for ABS-like bases). Carious lesions in the MSLA route were generated by manually injecting Flex 63A, a flexible photopolymer resin (Formfutura, Nijmegen, The Netherlands), into the pre-designed cavity volumes, simulating the soft consistency of necrotic dentin. Active operator time for cavity filling of a full arch is approximately 10–15 min.

### 2.8. Fabrication Route B: PolyJet Workflow

In the pure PolyJet route, the same digital model was printed on a Stratasys J5 DentaJet (Stratasys Ltd., Rehovot, Israel) [46] utilising the proprietary VeroDent material set [47] for a multi-material assignment across multiple anatomical, pathological, and restorative volumes. This setup featured an outer enamel shell, a dentin core, a pulp chamber, and simulated restorations (representing composite or amalgam), all sharing a uniform rigidity but differentiated by distinct colour profiles, alongside a dedicated carious lesion volume of altered mechanical properties.

**Figure 7:**
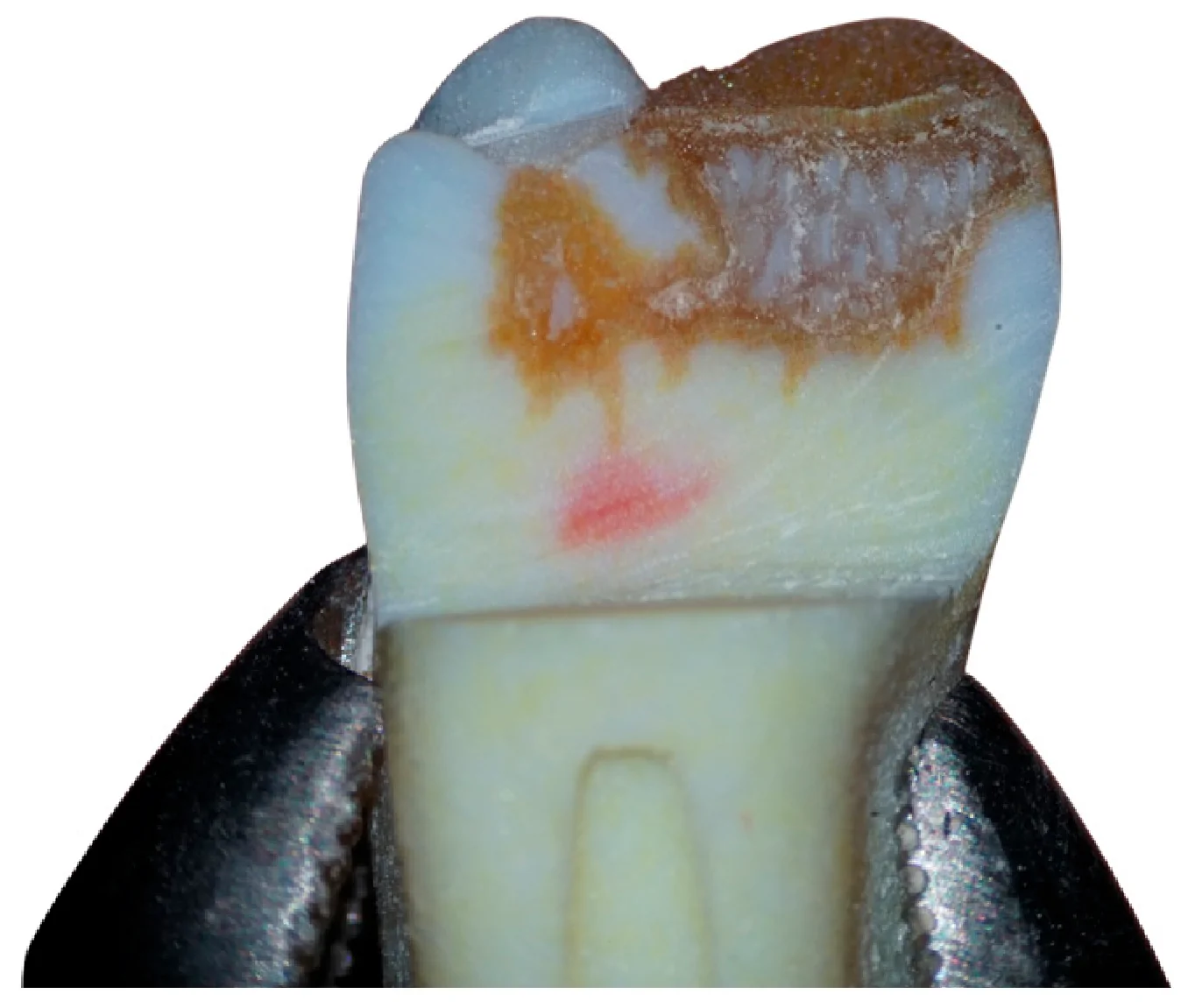
Cross-section through a multi-material PolyJet tooth, showing the enamel, dentin, pulp chamber and carious lesion, with the pulp chamber in point-contact with the lesion. A softer, lighter-coloured core is visible within the lesion, encapsulated in rigid resin.

**Figure 8:**
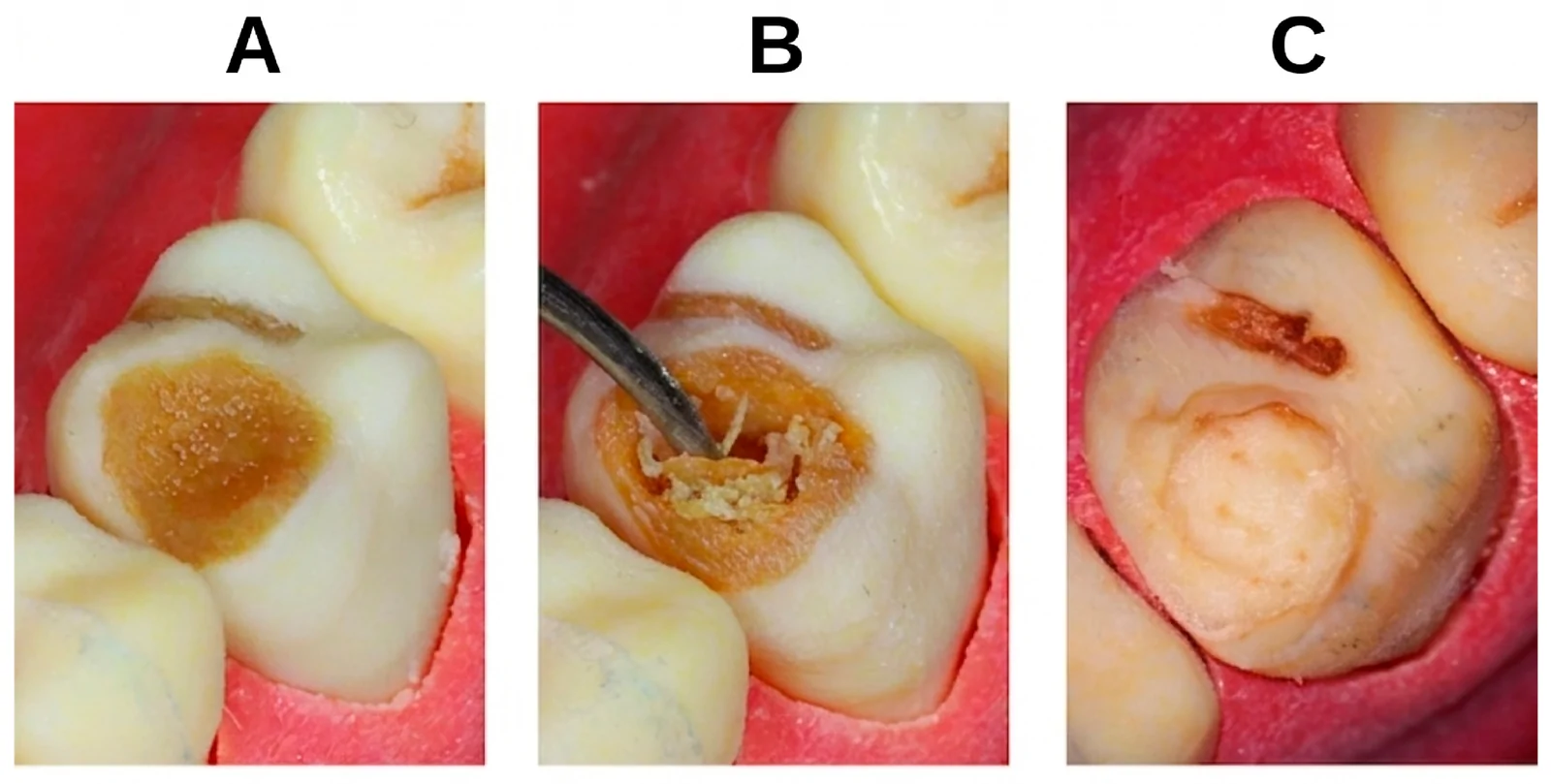
Simulated caries excavation on the PolyJet stratified tooth in operative use. (**A**) Occlusal carious lesion before instrumentation. (**B**) Mid-excavation, with softened carious tissue being removed. (**C**) Cavity after excavation: colour-coded affected dentin remains at the cavity floor, and a separate distopalatal-fossa lesion connects subsurface with the excavated lesion beneath the oblique enamel ridge.

The same lesion body that had previously been used as a Boolean subtraction tool against the enamel and dentin volumes (and which therefore exactly corresponds, by construction, to the negative space of the cavity) was hollowed to produce a thin closed shell of 0.2 mm wall thickness. In the slicer, the 0.2 mm shell was assigned to a rigid resin, while the enclosed internal volume was assigned to the soft PolyJet support material (SUP711S). The result is a thin rigid envelope encapsulating a soft core, occupying exactly the space of the clinical cavity (Figure 7). During student instrumentation, the bur first engages the thin rigid envelope, which offers a brief, brittle resistance reminiscent of demineralised enamel, and then enters the soft core, producing the “fall-through” tactile cue clinically associated with excavating softened dentin (Figure 8).

The PolyJet workflow is particularly well suited to batch fabrication. A single print run on the J5 DentaJet accommodates approximately 270–300 individual teeth, corresponding to 9–10 complete 30-tooth garnitures, without meaningful impact on print time. Post-processing is correspondingly batched: entire print batches, optionally accumulated from several successive runs, are left to soak in a water bath for several hours, up to overnight, during which the bulk of the gelatinous support matrix softens and separates spontaneously from the printed parts. The batch is then agitated and rinsed in a WaterJet station to remove the bulk of the softened support. Direct WaterJet impingement on the carious lesion zones is avoided to preserve the 0.2 mm protective shell. Because all steps are performed on batches rather than on individual models, active operator time per model is low and decreases further with batch size.

### 2.9. Fabrication Route C: Adopted Hybrid Workflow (MSLA Base + PolyJet Teeth)

The hybrid fabrication workflow was favoured a priori, on the basis of the comparative analysis of the candidate technologies, and was adopted as the principal production route of this study; the two pure routes described above were fabricated in parallel as benchmarks to validate this choice empirically. The hybrid allocates the two components of the model to the manufacturing technology best suited to each:

Model bases are produced on the MSLA line, using the protocol described in Section 2.7. The base is designed as a monolithic, uniform-material structural component whose fidelity requirements are modest (dimensional stability, mechanical rigidity, and accurate sockets); MSLA delivers these at minimal cost.

Tooth garnitures are produced on the Stratasys J5 DentaJet using the multi-material stratification and batched post-processing protocol described in Section 2.8. The tooth garniture is the component on which the student actually operates, where morphological, chromatic and haptic fidelity are all critical and where the multi-material capability of PolyJet delivers a qualitative advantage that MSLA cannot replicate.

This allocation has three practical advantages beyond component-level fidelity. First, it distributes wear across both machines: the PolyJet system, whose consumables and service costs are significantly higher, is not loaded with the production of bulky low-value base components, and its printing budget is concentrated on the small-volume high-value tooth garnitures. Second, it preserves the scalability of MSLA base production independently of PolyJet throughput, so that base stocks can be replenished asynchronously. Third, it decouples the two failure modes: a failed base print does not waste PolyJet resin, and a failed tooth batch does not block base production. In day-to-day operation, the hybrid route has been used successfully to produce the MK1 prototypes described in this work.

### 2.10. Final Assembly

**Figure 9:**
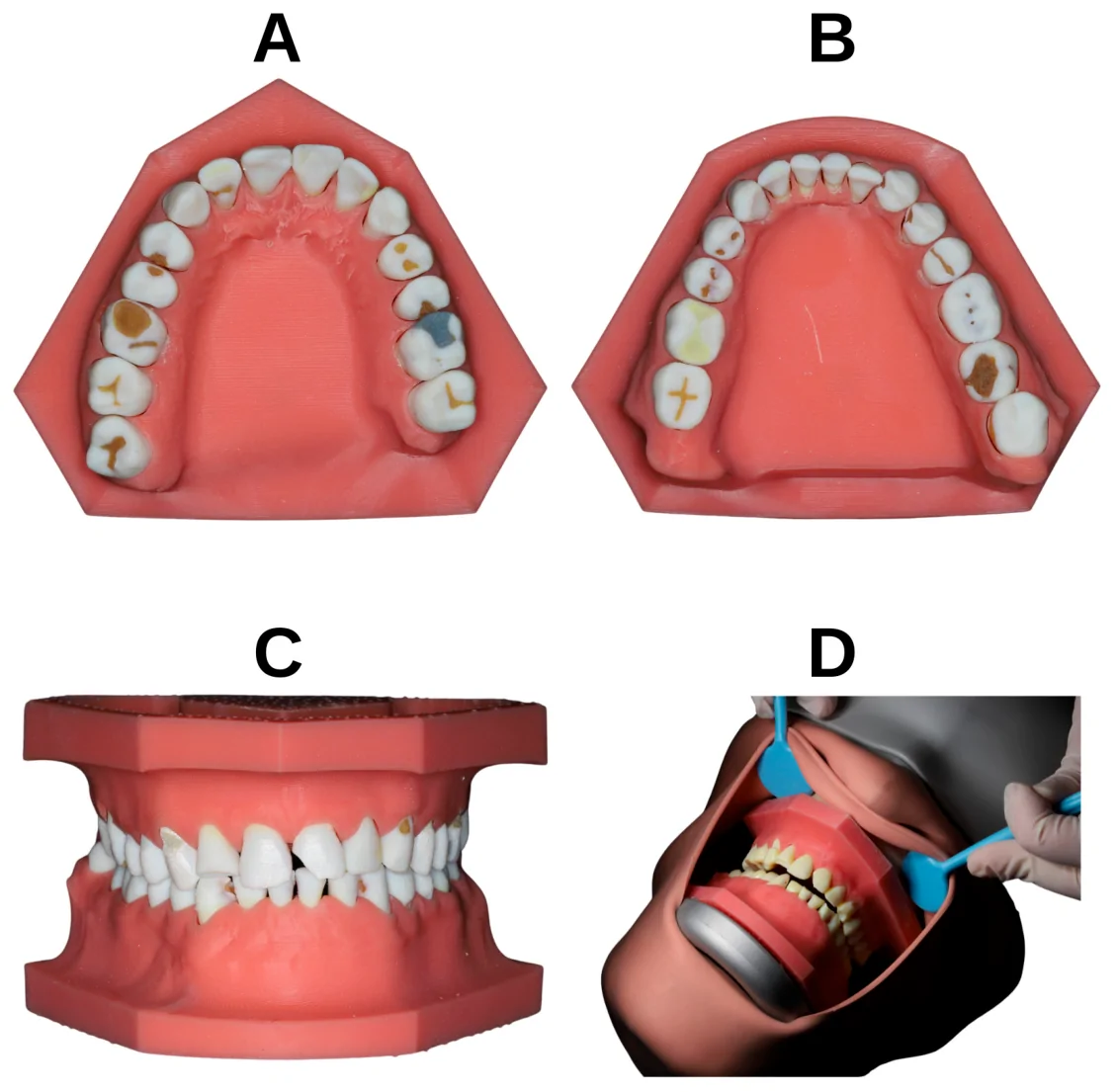
Final assembled MK1 prototype in the preclinical environment. (**A**) Occlusal view of the maxillary model. (**B**) Occlusal view of the mandibular model. (**C**) Complete bimaxillary assembly in occlusion, frontal view. (**D**) Models mounted on a Frasaco phantom head.

In all routes, the printed teeth were inserted into their corresponding sockets and secured by trans-apical screws driven from the base of the model. The anti-rotational cylindrical guides ensured a single deterministic seating, and the 3 mm basal floor provided sufficient anchorage for the metric screws. The complete assembly (Figure 9) is mounted on a Frasaco phantom head (Franz Sachs & Co./frasaco GmbH, Tettnang, Germany) via the M7 interface.

### 2.11. Evaluation of Perceived Fidelity and Didactic Value

Design and aim. A single-centre, cross-sectional questionnaire study was conducted to evaluate the learner’s perspective. The printed tooth was ranked against the two reference substrates students already know, the natural extracted tooth and the conventional plastic (acrylic) tooth, through categorical items rather than a separate side-by-side re-handling session.

Evaluation procedure. Each participant was provided with a PolyJet-printed stratified specimen of the MK1 prototype carrying simulated carious lesions, together with a dental explorer and the rotary instrumentation available in the preclinical laboratory. Participants were instructed to inspect the specimen visually, to engage the carious lesion with the explorer in order to appreciate its “soft” internal consistency, and to perform a complete simulated caries excavation followed by coronal restoration, following the standard clinical protocol taught in the curriculum. The questionnaire was completed online immediately after this hands-on evaluation.

A structured questionnaire was developed in Romanian and administered through Google Forms; it comprised two parts, a background section (Section A) and an evaluation section (Section B).

Section A collected five background items: sex, age group, highest completed level of education, area of residence, and the types of training substrate the respondent had previously worked on. The last was a multiple-response item on the substrate types previously used, from which prior experience with 3D-printed teeth (yes/no) was derived.

Section B comprised 22 items addressing the printed tooth. Thirteen were 5-point Likert items, each anchored individually to the construct being rated (for example 1 = “not at all realistic”, 5 = “identical to a natural tooth”). Four Likert items were deliberately reverse-worded, and three composite perception scores were defined a priori from the Likert items: a Visual Realism scale, a Tactile Realism scale and a Didactic Utility scale (suitability for cavity-preparation training, value of standardisation for repeated practice, and suitability for layered-restoration teaching).

The remaining nine items of Section B were categorical. Three single-choice items asked which of the three substrates (natural extracted, layered 3D-printed or conventional plastic tooth) offered the most realistic visual experience, the most realistic tactile experience, and which the respondent would prefer for preclinical practice. A five-level item compared the printed tooth against the natural extracted tooth as an overall training substrate, from “much worse” to “much better”. Further single-choice items addressed biological safety relative to extracted teeth, the most bothersome aspect encountered during preparation, and the perceived risk of acquiring clinically transferable incorrect habits. Two multiple-response items collected the perceived advantages of the printed tooth and the contexts in which respondents would not recommend its use.

Ethics and data handling. Participation was voluntary and anonymous; submission of the online form constituted informed consent, as stated in the questionnaire preamble, and no directly identifying information was collected.

### 2.12. Statistical Analysis

All analyses were performed in IBM SPSS Statistics 29.0.2.0. (IBM Corp., Armonk, NY, USA). Continuous and ordinal variables are summarised as mean (standard deviation), median (interquartile range) and, where appropriate, 95% confidence intervals; categorical variables are reported as frequencies and percentages. The internal consistency of each a priori composite scale was assessed with Cronbach’s coefficient α, values ≥ 0.70 being considered acceptable and values of 0.60–0.70 acceptable for exploratory research; corrected item–total correlations and the change in α expected on item removal were inspected. On this basis, the reverse-coded translucency item was excluded from the final Visual Realism scale, because its corrected item–total correlation was near zero and its removal raised α from 0.67 to 0.76; the revised five-item scale was retained for all subsequent analyses. The distribution of composite scores was examined with the Shapiro–Wilk test; because the Didactic Utility composite departed significantly from normality and all underlying items were ordinal, non-parametric methods were used throughout. Between-group comparisons used the Mann–Whitney U test, with prior experience with 3D-printed teeth (yes/no) as the grouping variable; the within-subjects comparison of the Visual and Tactile Realism composites used the Wilcoxon signed-rank test. Associations between categorical variables were examined with the Pearson chi-square test or, when expected cell counts fell below 5, the Fisher–Freeman–Halton exact test, with Cramér’s V as the effect-size measure. Two-tailed *p*-values < 0.05 were considered statistically significant. No a priori sample-size or power calculation was performed. The study was designed as an exploratory, descriptive evaluation of a newly developed prototype, and the analysis sample was a convenience cohort of eligible students available during the evaluation window rather than a target size derived from a hypothesised effect. A post hoc sensitivity analysis indicated that the study was well powered for its principal, pre-specified within-subject contrast: the observed Wilcoxon effect for Visual versus Tactile Realism was very large (r = |Z|/√N = 0.84), and a sample of fifty affords high power to detect effects of this magnitude. By contrast, the prior-experience subgroup comparison was constrained by the 40:10 group imbalance; at α = 0.05 and 80% power, this configuration can reliably detect only large effects (Cohen’s d ≈ 1.0), so the null subgroup results represent absence of evidence rather than evidence of absence. Because the study was exploratory and hypothesis-generating, *p*-values are reported without formal correction for multiple testing and should be interpreted accordingly; we note, however, that the single confirmatory within-subject finding (*p* < 0.001) survives any conventional multiplicity correction, whereas every subgroup comparison was already non-significant at the uncorrected level (smallest *p* = 0.108), so multiplicity does not account for any positive result reported here.

## 3. Results

### 3.1. Final Prototype

The fabrication protocol produced three complete bimaxillary “MK1” prototypes, one per fabrication route, mounted on standard Frasaco-compatible phantom heads. All prototypes preserved the source patient’s habitual occlusion, interproximal contact points and overall arch morphology, and exposed the full curricular spectrum of carious and non-carious lesions distributed across the two arches.

### 3.2. Geometric and Functional Assessment

On qualitative cross-sectional and functional assessment, the printed models reproduced the overall crown morphology and arch form of the source dentition, consistent with the documented accuracy of intraoral scanning and additive manufacturing for dental models [48,49,50,51]; a quantitative deviation analysis was not performed (Section 4.2). The combination of the zero-tolerance apical butt joint and the 0.2 mm lateral clearance constrained each tooth to a single deterministic vertical position corresponding to its location in the source digital dataset, so that the occlusal and interproximal contact points of the source dentition were reproduced on the physical prototype without articulating-paper refinement and reproducibly across successive insertion cycles. The trans-apical screw system, in combination with the convergent-cylinder anti-rotational mechanism, provided rigid intra-procedural fixation: teeth did not vibrate or rotate during simulated bur preparation, while remaining individually removable in seconds for inspection or replacement. The 0.2 mm circumferential clearance functioned simultaneously as a passive insertion tolerance and as a didactically meaningful gingival sulcus, allowing matrix band placement and subgingival instrumentation.

### 3.3. Multi-Material Stratification and Tactile Differentiation

Both fabrication routes produced tactilely differentiated carious tissue. In the MSLA route, the elastomeric resin manually injected into the pre-designed cavities offered a soft, yielding response distinct from the surrounding rigid C&B resin. In the PolyJet route, the three-layer digital material architecture (rigid enamel shell, coloured dentin core, soft support encapsulated under a 0.2 mm rigid envelope) produced the intended progressive haptic response on bur penetration. The anatomically variable enamel thickness was visually appreciable on cross-section, with cuspal regions displaying a thicker translucent layer than the cervical zones, consistent with the expected gradient of natural enamel.

### 3.4. Survey Participants

Fifty dental students completed the evaluation of the layered, PolyJet-printed 3D teeth, which were fitted into the model assembly and mounted inside a Frasaco phantom head to simulate a realistic patient scenario. (Table 1). Female respondents accounted for 64.0% of the sample, and all participants were in the 18–30 year age group. Almost all were undergraduates currently enrolled in the programme (96.0%), and most resided in urban areas (78.0%). Most participants had previously practised on several types of training substrate, including naturally extracted teeth (98.0%), conventional plastic teeth (96.0%) and non-stratified 3D-printed teeth (72.0%); only a small minority had previously worked on layered 3D-printed teeth (8.0%). Overall, 40 students (80.0%) reported some prior experience with 3D-printed teeth in either form, while 10 (20.0%) had none.

### 3.5. Item-Level Perceptions

**Figure 10:**
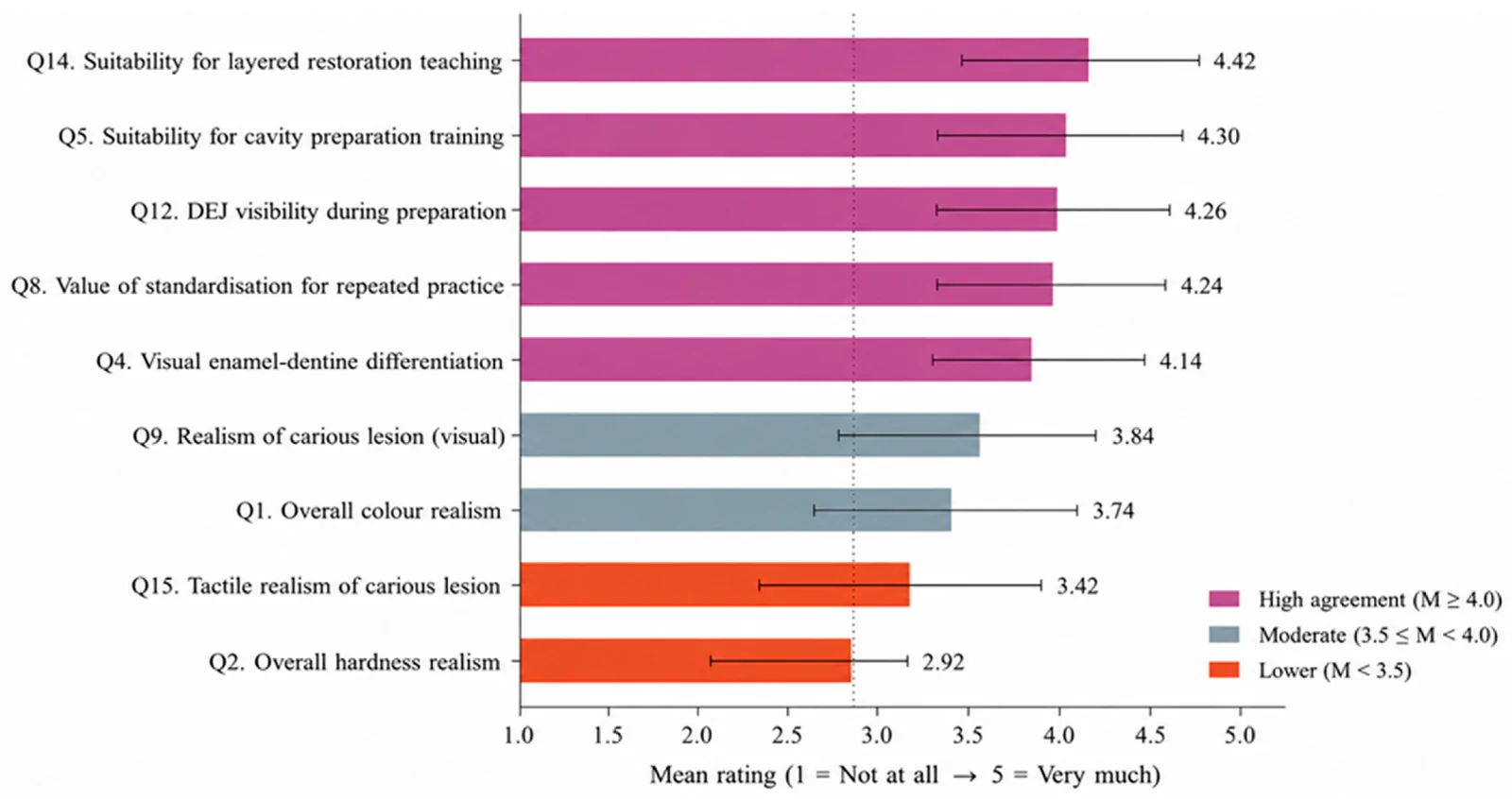
Mean (±SD) ratings on the 13 Likert items addressing perceptions of the layered 3D-printed tooth (*n* = 50). Items are ordered by mean rating; reverse-coded items are presented in their original direction.

Descriptive statistics for the thirteen Likert items are summarised in Table 2 and Figure 10. Items addressing didactic utility consistently attracted the highest endorsement: suitability for teaching layered composite restorations had the highest mean (M = 4.42, SD = 0.76), followed by suitability for cavity-preparation training (M = 4.30, SD = 0.81) and the value of standardisation for repeated practice (M = 4.24, SD = 0.94). Visual fidelity items were also rated favourably, with students perceiving a clear visual differentiation between enamel and dentine (M = 4.14, SD = 0.99), good dentino-enamel junction visibility during preparation (M = 4.26, SD = 0.88) and acceptable colour realism (M = 3.74, SD = 1.01). Tactile items produced markedly lower scores: overall hardness realism had the lowest mean of all items (M = 2.96, SD = 0.86), and tactile realism of the carious lesion was rated only moderately (M = 3.42, SD = 0.97). The reverse-coded item on the carious lesion feeling like an empty cavity had a mean of 1.98 (SD = 0.96) in its original direction, indicating that 72% of respondents disagreed with that negative statement.

### 3.6. Composite Scores and Scale Reliability

**Figure 11:**
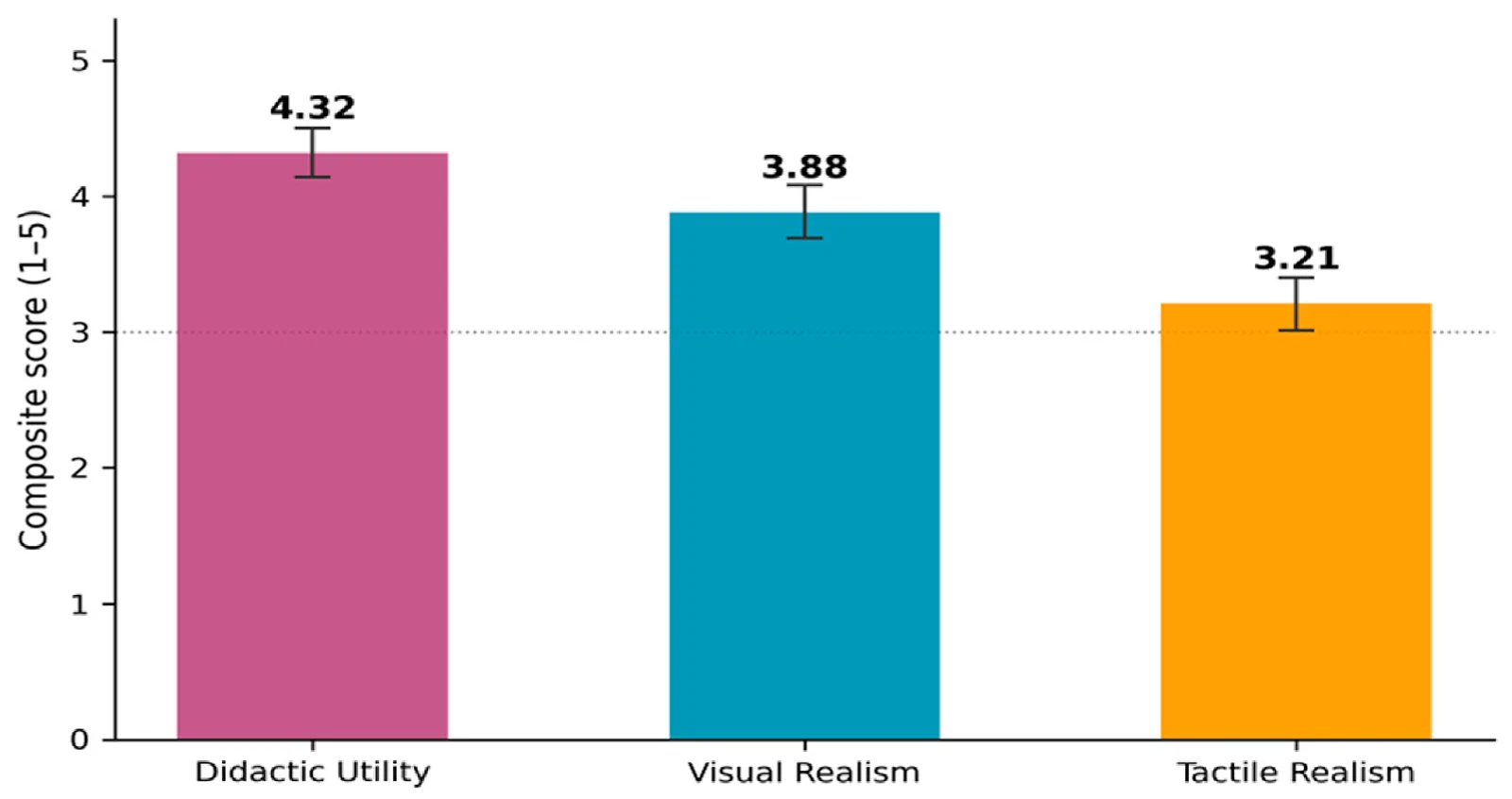
Composite perception scores (means with 95% confidence intervals). The dotted line indicates the neutral midpoint of the rating scale (=3).

The three composite scores demonstrated acceptable internal consistency (Cronbach’s α between 0.65 and 0.76; Table 3). Didactic Utility was rated highest (M = 4.32, SD = 0.65; Mdn = 4.50), followed by Visual Realism (M = 3.88, SD = 0.69; Mdn = 3.90) and Tactile Realism (M = 3.21, SD = 0.68; Mdn = 3.25); composite scores are visualised in Figure 11. A Wilcoxon signed-rank test confirmed that Visual Realism was rated significantly higher than Tactile Realism within the same respondents (Z = −5.91, *p* < 0.001).

### 3.7. Categorical Preferences and Overall Evaluation

**Figure 12:**
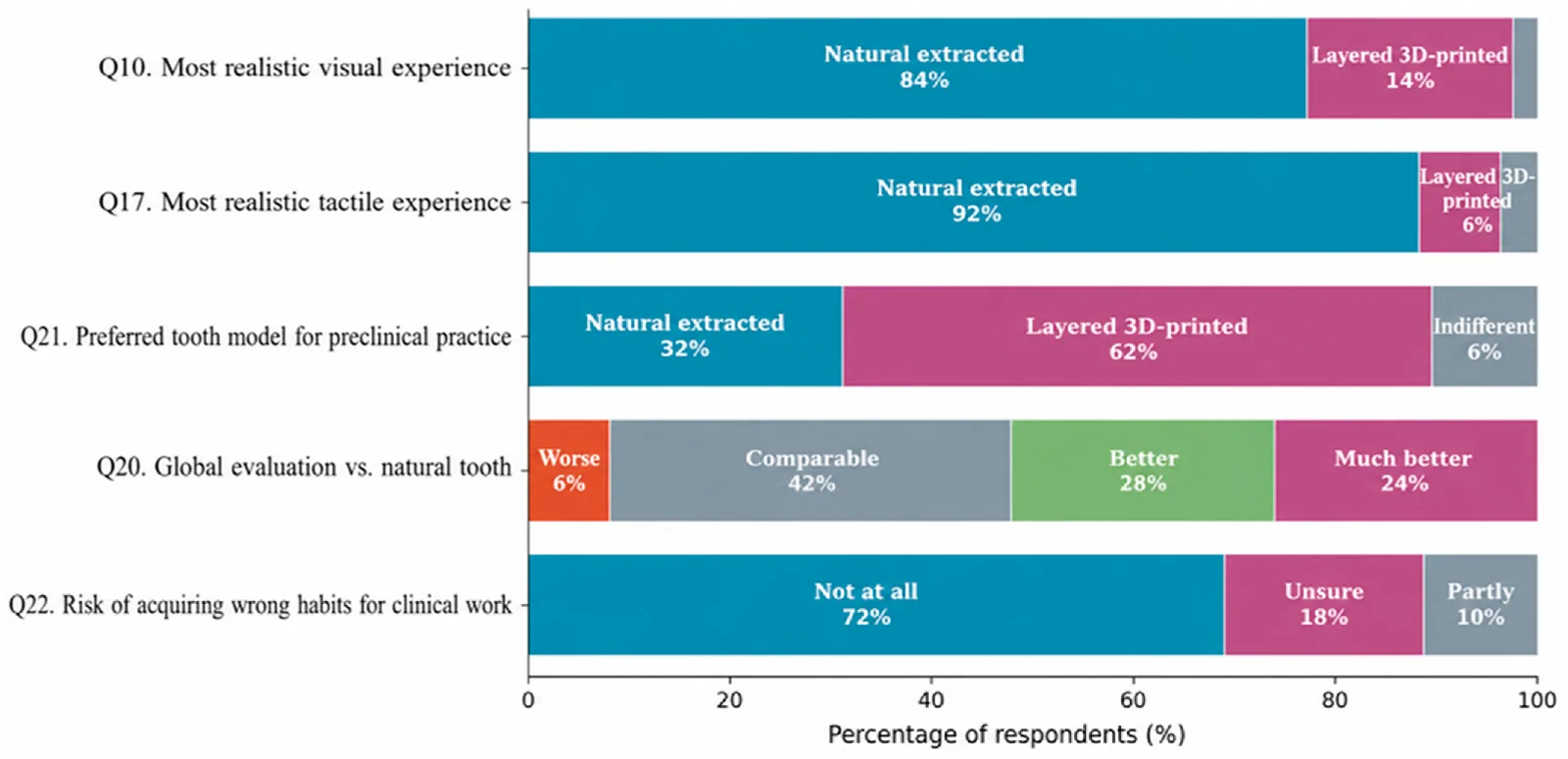
Distribution of responses on five categorical items.

The distribution of responses for the main categorical items is presented in Figure 12. When asked which substrate offers the most realistic visual experience, 84% of students selected the natural extracted tooth, 14% the layered 3D-printed tooth and 2% reported being unable to differentiate between them. For the tactile experience, 92% favoured the natural extracted tooth, 6% the layered 3D-printed tooth and 2% the conventional plastic tooth. On the five-level global comparison item, 94% of students rated the printed tooth as at least comparable to the natural tooth (42% comparable, 28% better, 24% much better), with only 6% rating it as worse. When asked which model they would prefer for preclinical practice if free to choose, 62% selected the layered 3D-printed tooth, 32% the natural extracted tooth and 6% expressed no preference. On the biological-safety item, 74% of respondents considered the printed tooth much safer than the natural extracted tooth, and a further 24% considered it safer, for 98% favourable responses overall. On the item addressing the risk of acquiring incorrect habits transferable to clinical practice, 72% answered “not at all”, 18% were unsure, and 10% acknowledged a partial risk.

### 3.8. Perceived Advantages and Disadvantages

**Figure 13:**
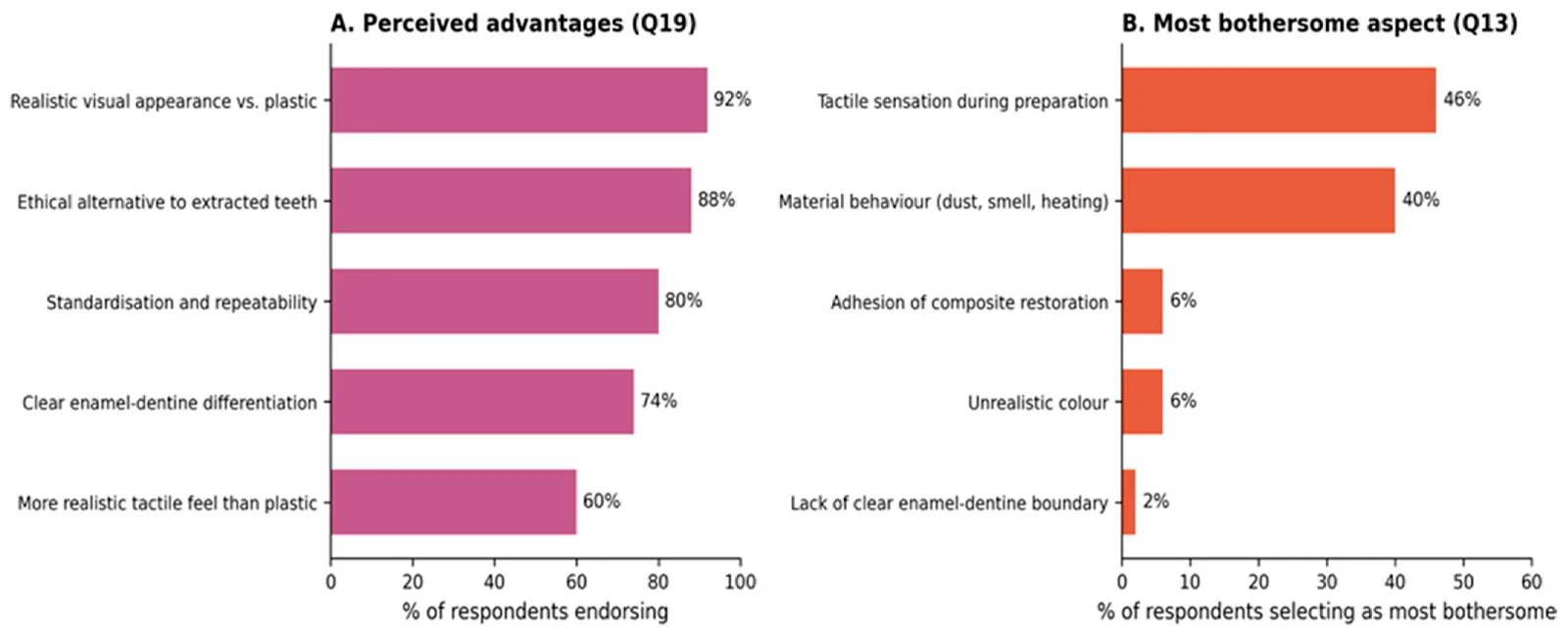
(**A**) Perceived advantages of the layered 3D-printed tooth (multi-response). (**B**) Most bothersome aspect during preparation (single response) (*n* = 50).

Figure 13 summarises the multi-response item on perceived advantages and the single-response item on the most bothersome aspect during preparation. The most frequently endorsed advantages were a more realistic visual appearance than plastic (92%), an ethical alternative to extracted teeth (88%), standardisation and repeatability (80%) and a clear enamel–dentine differentiation during preparation (74%); a more realistic tactile sensation than conventional plastic was endorsed by 60% of respondents. The most bothersome aspects related to tactile and material behaviour rather than to visual features: 46% of students identified the tactile sensation during preparation as the most disturbing element, followed by the behaviour of the printed material at the bur (dust, odour, heating) at 40%, while composite adhesion (6%), unrealistic colour (6%) and lack of a clear DEJ (2%) were rarely cited. Regarding contexts in which the layered 3D-printed tooth should not be used, 76% of students would recommend it in all preclinical contexts; among the remaining 24%, the most frequent reservations concerned fine aesthetic restorations (12%) and the very first years of study (12%), with examination contexts (6%) and adhesive-technique training (4%) rarely cited.

### 3.9. Influence of Prior Experience with 3D-Printed Teeth

**Figure 14:**
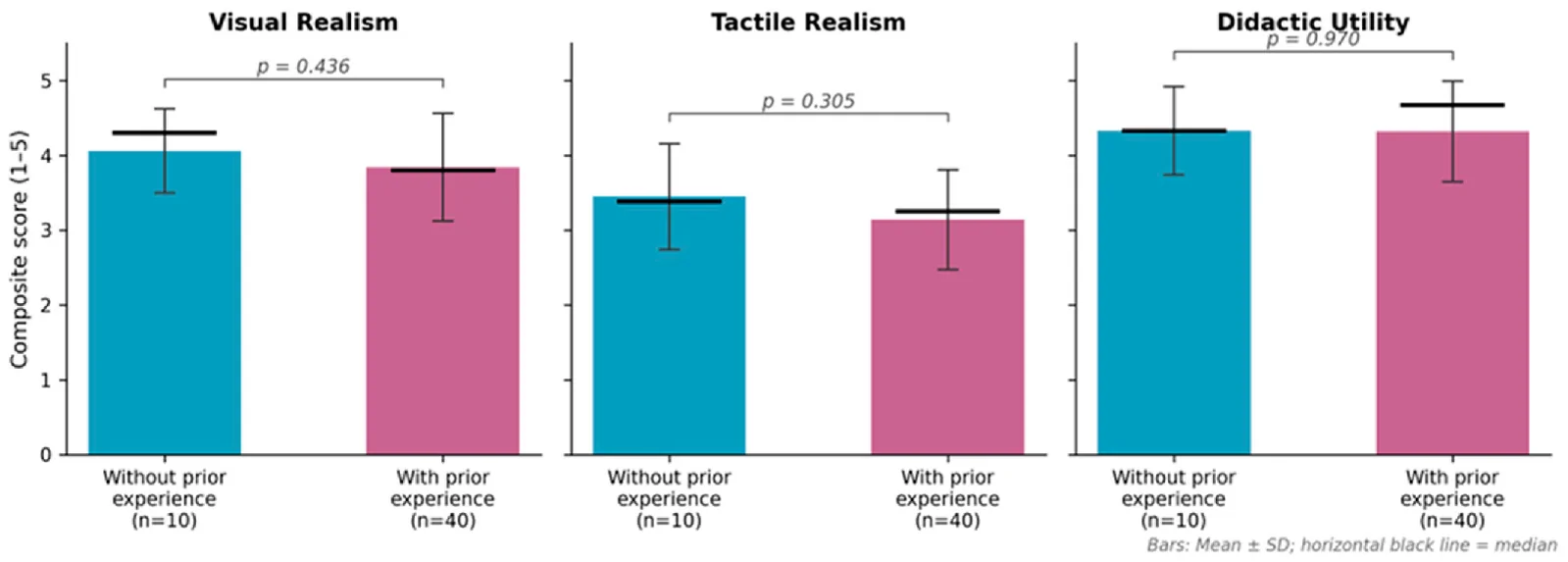
Composite perception scores by prior experience with 3D-printed teeth. Bars represent means ± SD; horizontal lines indicate group medians. *p*-values are from Mann–Whitney U tests.

Forty students (80%) reported prior experience with 3D-printed teeth in any form, while ten (20%) had none. Mann–Whitney U tests comparing the two groups on composite scores and on the main Likert items revealed no statistically significant differences (Table 4, Figure 14). Composite Visual Realism was, on average, slightly higher among inexperienced students (M = 4.06, SD = 0.56) than experienced ones (M = 3.84, SD = 0.72), but the difference was not significant (U = 168.0, Z = −0.78, *p* = 0.436); similar non-significant patterns were observed for Tactile Realism (U = 158.0, Z = −1.03, *p* = 0.305) and Didactic Utility (U = 198.5, Z = −0.04, *p* = 0.970). The largest item-level difference appeared for overall colour realism, rated somewhat higher by inexperienced students (means 4.20 vs. 3.62), but this difference too remained below the conventional threshold of significance (*p* = 0.108). Crosstabulation with the Fisher–Freeman–Halton exact test revealed no significant association between prior experience and the categorical outcomes (most realistic visual experience, *p* = 0.312; most realistic tactile experience, *p* = 1.000; global evaluation, *p* = 0.906; preferred model, *p* = 0.857; risk of incorrect habits, *p* = 0.114). Given the marked imbalance between subgroups (*n* = 10 vs. *n* = 40), these comparisons should be interpreted as exploratory and underpowered.

## 4. Discussion

### 4.1. Significance of the Proposed Workflow

The present work describes a reproducible end-to-end digital workflow for the in-house fabrication of patient-specific multi-material dental simulators that simultaneously address the three principal limitations of previously published 3D-printed educational models: monolithic single-material prints, simplified retention geometry and colour-only caries representation [13,14,16,25]. Because the entire model is derived from real intraoral and CBCT data of a single curated case, it reproduces the inter-tooth variability, asymmetries and contact-point geometry of an actual dentition while remaining infinitely reproducible, combining the anatomical fidelity of natural teeth with the inter-student standardisation required for fair assessment, an objective that conventional typodonts achieve only through excessive idealisation and VR/haptic systems at prohibitive cost. A specific consequence of this approach is the accurate reproduction of the source dentition’s occlusal and interproximal contact points in the physical prototype. Because the tooth seats rigidly against a zero-tolerance apical stop at exactly the vertical position prescribed by the source data, the contact geometry is inherited directly from the source dentition, instead of being re-created afterwards by adjusting the teeth on an articulator. For procedures in which contact relationships are the principal didactic target (interproximal matrix placement, proximal box preparations, contact restoration with composite, occlusal anatomy reconstruction), this property of the modular architecture is arguably as important as the morphological fidelity of the individual teeth.

The modular architecture reconciles three requirements usually met only in part: rigid intra-procedural stability, repeatable replacement of individual teeth at marginal cost, and a clinically realistic gingival sulcus accessible to subgingival instrumentation.

Anatomically driven enamel stratification and mechanically differentiated caries. Multilayer printed teeth with separate enamel and dentin have been described before [24], but the enamel layer was generated as a uniform-thickness offset. The variable-thickness shell introduced here is, to our knowledge, the first to derive enamel stratification directly from the tooth geometry itself. As described in Section 2.5, this protocol produces an anatomically oriented gradient of enamel thickness from a single fixed parameter set applied identically to every tooth. As a result, any cross-section through the printed tooth presents students with an enamel-dentin-pulp stratigraphy that qualitatively resembles the one they encounter on extracted teeth and clinical radiographs. This anatomically oriented internal architecture is, in our view, the single most important advance over monolithic printed teeth, and it is obtained at essentially no additional design cost once the protocol has been established.

The caries architecture reuses the same lesion body, so that in the PolyJet route a thin brittle envelope over a soft core is placed exactly at the clinical lesion site with no further design effort, producing on bur contact the brief resistance and subsequent “fall-through” cue clinically associated with excavating softened dentin (Section 2.8). The MSLA equivalent reaches a comparable haptic outcome by manual elastomeric resin injection, at the cost of greater operator time.

The hybrid allocation was favoured a priori, on the basis of a cost-function analysis of the two technologies, and the two pure routes were fabricated in parallel chiefly to test this choice empirically. The comparison confirmed the underlying rationale: the consumable cost of the pure PolyJet route is driven by the proprietary resin needed for the bulky model bases, not by the tooth garniture, so assigning the base to MSLA removes the principal cost driver while retaining PolyJet only for the component whose multi-material capability MSLA cannot replicate (Section 2.9). In absolute terms, the hybrid model is produced at a consumable cost of approximately 12 to 13 EUR per complete bimaxillary set, comparable to the pure MSLA route (approximately 12 EUR) and roughly one quarter of the pure PolyJet route (approximately 50 EUR); relative to an equivalent commercial model (on the order of 112 EUR [52]), this represents an approximately 90% reduction in per-model consumable cost (a comparison of in-house consumable expenditure against a commercial retail price, excluding printer acquisition and depreciation, maintenance, and operator labour). All cost figures reported in this study reflect consumable and material expenditure only; a full cost-of-ownership analysis incorporating capital equipment, depreciation, maintenance, and labour was outside the scope of the present comparison. The pure MSLA route remains a fully valid fallback for schools without PolyJet access. The pure PolyJet route remains defensible in niche contexts where the additional cost is absorbed by the specific use case, or where the presence of the trans-apical screw is incompatible with the intended didactic objective, as in the dedicated endodontic branch of the model family described below.

Root canals were deliberately omitted from the MK1 model because the trans-apical screw, the feature that makes individual tooth replacement economical, occupies the apical third of each root and is incompatible with a patent canal (Section 2.5). Endodontic competence depends on managing complex root canal anatomy that is frequently missed and a recognised contributor to treatment failure [53]; to address this need, a separate model branch was developed in parallel, designed with dedicated features for endodontic practice. A dedicated description of the endodontic branch is beyond the scope of the present paper and will be reported separately.

The entire CAD pipeline is built on freely available or low-cost software, unlike the locked ecosystems of proprietary CAD/CAM suites. Combined with the M7 interface compatibility, this means the workflow can be replicated in any dental school with access to a basic MSLA printer, without dependence on proprietary licensing; the hybrid and pure-PolyJet routes additionally require a multi-material PolyJet system, a capital investment not universally available, and are therefore positioned for institutions that already operate such hardware. This may be regarded as a precondition for moving 3D-printed simulators from academic curiosity to standard curricular infrastructure. Because intraoral scanners and MSLA/PolyJet printers are increasingly part of standard digital-dentistry teaching infrastructure, for institutions where this equipment is already available the incremental cost of adopting the workflow is dominated by consumables, which is the sense in which the present figures are reported.

Second, the printed tooth was perceived as more realistic visually than tactually. Hardness realism received the lowest score (M = 2.96), mainly due to the uniform mechanical properties of the printed material. Although the optical appearance was convincing, current PolyJet materials do not yet replicate the mechanical heterogeneity of natural enamel and dentin, consistent with previous studies [5,6,54].

Third, despite their tactile limitations, layered 3D-printed teeth were preferred for preclinical training by 62% of students, compared with 32% for extracted teeth. This preference was supported by perceived biological safety (98%) and standardisation (80%), suggesting that students value these advantages alongside realism. Moreover, 72% did not believe that training on printed teeth would promote incorrect clinical habits, consistent with previous studies [5,52,55,56].

Prior experience with 3D-printed teeth did not significantly modulate perceptions in this sample. Students without prior experience rated all three composites slightly more favourably than experienced students, but none of the comparisons reached statistical significance, and the differences were small. Either familiarity does not substantially shift perception (students may simply react to the artefact in front of them) or the study was underpowered to detect such modulation, given the marked imbalance between the experienced (n = 40) and inexperienced (n = 10) subgroups; a larger, more balanced sample would be needed to resolve this. Taken together, the survey indicates that the PolyJet-printed stratified tooth is perceived by students as didactically useful, visually convincing and ethically preferable to extracted teeth, and that a majority would adopt it for preclinical practice, while the tactile dimension, particularly perceived hardness and material behaviour during preparation, remains the principal limitation to be addressed by continued material development.

### 4.2. Limitations

First, the workflow was developed on a single source case; generalisation to atypical anatomies (severe crowding, partial edentulism, marked periodontal bone loss) will require additional curated source datasets. Second, the mechanical behaviour of the printed materials was characterised only from the learner’s perspective and not by instrumented testing. The substrates are commercially available formulations, but their properties as deployed in the present multi-material construct were not independently verified, and the haptic differentiation between sound and carious tissue was not quantified by instrumented measurement of cutting forces. Third, the enamel-shell protocol reproduces the anatomically oriented thickness gradient of natural enamel (thicker over the cusps and incisal edges, thinning toward the cervical margin) as an emergent geometric consequence of the smoothing operation, rather than a thickness targeted to a specified value. Furthermore, the geometric fidelity of the printed models to the source digital dataset was assessed only qualitatively and was not quantified by a formal STL-to-print deviation analysis; an independent metrological validation of dimensional accuracy across the three fabrication routes was outside the scope of the present proof-of-workflow study and is planned as a next step. Fourth, by design, the restorative MK1 model does not contain patent root canals, because the trans-apical retention system is mechanically incompatible with them. Fifth, the perceptual evaluation has its own constraints: it was a single-centre convenience sample of 50 students of relatively homogeneous age and seniority, with a marked imbalance between students with and without prior 3D-printing experience (40 vs. 10), which limits external validity and the power of the subgroup analyses; self-report bias is inherent to attitudinal surveys, only partially mitigated by the reverse-coded items. Sixth, perceptions were not paired with objective performance measures, so the study documents user acceptance and perceived didactic value rather than educational effectiveness and cannot establish whether the favourable perceptions translate into measurable skill acquisition. In addition, no a priori sample-size or power calculation was performed, in line with the exploratory aim of this first evaluation; sample size was set pragmatically by student availability. A post hoc sensitivity analysis confirmed adequate power for the principal within-subject contrast (very large effect, r = 0.84) but not for the prior-experience subgroup comparison, where the 40:10 split allows detection of only large effects (d ≈ 1.0). Finally, multiple comparisons are reported without multiplicity correction; this does not affect our conclusions, since the sole confirmatory finding (Visual > Tactile Realism, *p* < 0.001) is robust to any standard correction and all subgroup comparisons were already non-significant. The subgroup findings should thus be read as hypothesis-generating, pending a larger, a priori-powered study.

### 4.3. Future Directions

Ongoing developments include the integration of a curated case library spanning the full spectrum of restorative scenarios, a structured interdisciplinary protocol for defining case-specific learning objectives with departmental faculty, the exploration of augmented-reality overlays providing real-time feedback on cavity-preparation depth, and, most directly motivated by the present findings, the development of resin formulations with differential hardness across the enamel and dentine layers, together with a longitudinal controlled study comparing preclinical performance and clinical transfer between students trained on different substrates. A further extension is the generation of synthetic radiographic counterparts in which the modelled lesion library is explicitly encoded into the imaging volume, so that each physical lesion on the model corresponds to a matching radiolucency on the radiograph; this would allow trainees to practise radiographic–clinical correlation, predicting a lesion from its radiographic appearance and verifying it on the physical substrate.

## 5. Conclusions

A reproducible, low-cost digital workflow has been developed for the in-house fabrication of patient-specific, multi-material 3D-printed dental simulators starting from routinely available IOS and CBCT data.

The proposed modular tooth-socket architecture (combining truncated-conical roots, a convergent-cylinder anti-rotational system, trans-apical screw retention and a 0.2 mm circumferential sulcus offset) provides rigid intra-procedural stability while enabling individual tooth replacement at <0.5 EUR per unit.

Anatomically variable enamel stratification, combined with the indirect (negative-volume) lesion modelling method and multi-material assignment, allows the resulting prototypes to present an anatomically oriented enamel-dentin-pulp-lesion stratigraphy on cross-section and to provide a tactilely differentiated response to instrumentation.

The hybrid MSLA/PolyJet route was adopted as the principal production workflow, combining the economic advantage of MSLA for the model base with the multi-material fidelity of PolyJet for the stratified teeth, at approximately 12 to 13 EUR per complete bimaxillary model, an approximately 90% reduction relative to an equivalent commercial reference. The pure MSLA route remains a low-cost fallback for institutions without PolyJet access, and the pure PolyJet route is reserved for niche or high-stakes applications; individual teeth remain replaceable at marginal cost (approximately 8 EUR per 30-tooth garniture). The layered 3D-printed tooth is best positioned as a standardised, ethically defensible adjunct to naturally extracted teeth in preclinical training, with material development on differential hardness as the priority next step.

In-house additive manufacturing of patient-specific dental educational models is feasible and well received by learners as a practical, deployable workflow for preclinical operative dentistry training; the present data establish feasibility and favourable user perception rather than superiority over existing systems, and confirmation of educational impact through performance-based studies remains a necessary step before the approach can be considered standard curricular infrastructure.

## Figures and Tables

**Table 1:** Sociodemographic characteristics and prior practice substrates of the sample (*n* = 50).

Characteristic	*n*	%
**Sex**		
Female	32	64.0
Male	18	36.0
**Age group**		
18–30 years	50	100.0
**Educational level**		
Undergraduate, in progress	48	96.0
Undergraduate, completed	2	4.0
**Residence**		
Urban	39	78.0
Rural	11	22.0
**Prior practice substrates (multi-response)**		
Natural extracted teeth	49	98.0
Plastic (acrylic) teeth	48	96.0
Non-stratified 3D-printed teeth	36	72.0
Layered (stratified) 3D-printed teeth	4	8.0
**Any prior experience with 3D-printed teeth**		
Yes	40	80.0
No	10	20.0

**Table 2:** Descriptive statistics for the 13 Likert items addressing perceptions of the layered 3D-printed tooth (*n* = 50). Items marked (R) are reverse-worded; values are shown in their original direction.

Item	M	SD	Mdn	Mode	*n*
Q1. Realism of overall colour	3.74	1.01	4	4	50
Q2. Realism of overall hardness	2.96	0.86	3	3	50
Q3. Enamel layer appears opaque (R)	3.10	1.02	3	3	50
Q4. Visual enamel–dentine differentiation	4.14	0.99	4	5	50
Q5. Suitability for cavity preparation training	4.30	0.81	4.5	5	50
Q6. Artificial appearance (R)	2.56	1.03	3	3	50
Q8. Standardisation for repeated practice	4.24	0.94	5	5	50
Q9. Realism of carious lesion (visual)	3.84	0.92	4	4	49
Q11. Behaves like plastic during preparation (R)	3.58	0.95	4	4	50
Q12. DEJ visibility during preparation	4.26	0.88	4.5	5	50
Q14. Suitability for layered restoration teaching	4.42	0.76	5	5	50
Q15. Tactile realism of carious lesion	3.42	0.97	3	3	50
Q16. Caries feels like an empty cavity (R)	1.98	0.96	2	1	50

**Table 3:** Composite perception scores: number of items (k), Cronbach’s α, central tendency, dispersion, 95% confidence interval for the mean, and Shapiro–Wilk test for normality (*n* = 50).

Composite Score	k	α	M (SD)	Mdn (IQR)	95% CI	S–W *p*
Visual Realism (without Q3)	5	0.76	3.88 (0.69)	3.90 (1.00)	[3.69, 4.07]	0.253
Tactile Realism	4	0.71	3.21 (0.68)	3.25 (0.94)	[3.01, 3.40]	0.548
Didactic Utility	3	0.65	4.32 (0.65)	4.50 (1.25)	[4.14, 4.50]	<0.001

**Table 4:** Comparison of composite scores and key items between participants with and without prior experience using 3D-printed teeth (Mann–Whitney U tests).

Variable	No Prior Exp. (*n* = 10) M (SD); Mdn	with Prior Exp. (*n* = 40) M (SD); Mdn	U	Z	*p*
Visual Realism (composite)	4.06 (0.56); 4.30	3.84 (0.72); 3.80	168.0	−0.78	0.436
Tactile Realism (composite)	3.45 (0.71); 3.38	3.14 (0.67); 3.25	158.0	−1.03	0.305
Didactic Utility (composite)	4.33 (0.59); 4.33	4.32 (0.67); 4.67	198.5	−0.04	0.970
Q1. Overall colour realism	4.20; 4.00	3.62; 4.00	137.5	−1.61	0.108
Q2. Overall hardness realism	3.00; 3.00	2.95; 3.00	191.0	−0.23	0.815
Q4. Enamel–dentine differentiation	4.30; 4.00	4.10; 4.00	191.0	−0.23	0.815
Q5. Suitability cavity preparation	4.40; 4.50	4.28; 4.50	189.5	−0.28	0.781
Q8. Standardisation value	4.30; 4.50	4.22; 5.00	198.5	−0.04	0.968
Q9. Carious lesion (visual)	4.20; 4.00	3.74; 4.00	143.0	−1.36	0.175
Q12. DEJ visibility	4.10; 4.00	4.30; 5.00	170.5	−0.78	0.436
Q14. Layered restoration teaching	4.30; 4.50	4.45; 5.00	179.0	−0.57	0.567
Q15. Tactile realism of caries	3.80; 4.00	3.33; 3.00	142.5	−1.46	0.143

Note. U = Mann–Whitney U statistic; Z = standardised test statistic; *p* = asymptotic two-tailed significance.

## Data Availability

The original contributions presented in the study are included in the article; further inquiries can be directed to the corresponding authors.

## Supplementary Materials

The following supporting information can be downloaded at: https://www.mdpi.com/article/10.3390/jfb17080355/s1, Table S1. Quantitative summary of the MK1 lesion rubric by clinical category.

## Abbreviations

CAD/CAM	Computer-aided design/computer-aided manufacturing
CBCT	Cone-beam computed tomography
C&B	Crown-and-bridge (resin)
CI	Confidence interval
CEJ	Cemento-enamel junction
DEJ	Dentino-enamel junction
DICOM	Digital Imaging and Communications in Medicine
GDPR	General Data Protection Regulation
IOS	Intraoral scan/intraoral scanner
IPA	Isopropyl alcohol
IQR	Interquartile range
ISO	International Organisation for Standardisation
MSLA	Masked stereolithography
RMS	Root mean square
SPSS	Statistical Package for the Social Sciences
STL	Standard Tessellation Language
