# Supplementary material for: A Reproducible Digital Workflow for Patient-Specific 3D-Printed Teeth with Anatomically Stratified Enamel and Multi-Material Caries for Preclinical Operative Dentistry
Source: J Funct Biomater. 2026 Jul 24;17(8):355. doi: 10.3390/jfb17080355 (PMC13514268; doi:10.3390/jfb17080355)
Supplement: Supplementary file 1 [file jfb-17-00355-s001.zip › jfb-4414693-supplementary.pdf]

Table S1. Quantitative summary of the MK1 lesion rubric by clinical category.

**Clinical category**	**No. of lesions**	**Teeth (FDI)**
Primary caries, Class I	8	1.2, 1.7, 1.8, 2.4, 2.7, 3.4, 3.7, 4.7
Primary caries, Class II	7	1.4, 1.5, 2.5, 3.5, 4.4, 4.5, 4.6
Primary caries, Class III	3	1.1, 3.2, 3.3
Primary caries, Class IV	3	3.1, 4.1, 4.3
Primary caries, Class V	3	2.6, 3.5, 4.8
Restorations with secondary caries	3	1.6, 2.2, 3.6
Pulpal involvement (exposure)	1	4.6
Non-carious lesions (substance loss and trauma) [1]	6	1.2, 1.3, 2.1, 2.3, 3.2, 4.2
**TOTAL**	**34**	

[1] The non-carious category comprises five substance-loss lesions (cervical wedge-shaped/abfraction, attrition and abrasion) and one non-penetrating mesial-angle fracture of a maxillary incisor (tooth 2.1). Four teeth carry two lesions each (1.2, 3.2, 3.5, 4.6), so the 34 lesions are distributed across 30 teeth.
